# Identification of bronchiolitis profiles in Italian children through the application of latent class analysis

**DOI:** 10.1186/s13052-020-00914-4

**Published:** 2020-10-07

**Authors:** Giuliana Ferrante, Carmela Fondacaro, Giovanna Cilluffo, Piera Dones, Francesca Cardella, Giovanni Corsello

**Affiliations:** 1 Department of Health Promotion Sciences Maternal and Infant Care, Internal Medicine and Medical Specialities, University of Palermo, Via del Vespro 129, 90127 Palermo, Italy; 2 National Research Council of Italy, Institute for Biomedical Research and Innovation, Via Ugo La Malfa 153, 90146 Palermo, Italy; 3 Pediatric Infectious Diseases Unit, ARNAS Civico Di Cristina, Via Benedettini 1, 90134 Palermo, Italy; 4 Department of Pediatrics, ARNAS Civico Di Cristina, Via Benedettini 1, 90134 Palermo, Italy

**Keywords:** Bronchiolitis, Children, Latent class analysis, Respiratory syncytial virus

## Abstract

**Background:**

Bronchiolitis is the primary infection of the lower respiratory tract in children under 2 years of age. Although it is generally considered a single nosological entity, recent studies suggested remarkable clinical heterogeneity. To date, no studies have identified classes of children with bronchiolitis within the Italian population. This study aimed to identify discrete profiles of Italian children hospitalized with bronchiolitis using a clustering approach and to compare findings with those obtained in international cohorts.

**Methods:**

This was a retrospective single-centre study conducted on children aged ≤2 years hospitalised with bronchiolitis (*n* = 401) at the Department of Infectious Diseases and the University Department of General Pediatrics in “Giovanni Di Cristina” Pediatric Hospital of Palermo, Italy, between November 2012 and May 2019. Bronchiolitis profiles were determined by latent class analysis, classifying children based on clinical characteristics at admission and viral aetiology.

**Results:**

Three profiles were identified. Class 1 (49%) was composed of 45% male children; all children were aged ≤6 months at hospitalization; 77% were infected with RSV; 100% had respiratory distress, 11% had apnea and none had cough. Class 2 (77%) was mainly composed of male subjects (51%); 19% were aged > 6 months at admission; 37% were infected with RSV; 12% had respiratory distress, 5% had apnea and 90% had cough. Class 3 (19%) included the largest proportion of male subjects (94%) and was mostly composed of children aged > 6 months at the time of admission (68%); 70% had cough, 12% showed respiratory distress and none presented with apnoea. Children in Class 1 were more frequently born near the epidemic season (*p* = 0.028); breastfeeding duration was significantly longer for children in Class 3 (*p* = 0.004).

**Conclusions:**

The study identified distinct clinical profiles of bronchiolitis by a clustering approach in a single-centre study of children hospitalised for bronchiolitis in Italy. The three bronchiolitis profiles share some similarities with those identified in international studies using the same statistical approach. These findings may help to increase the understanding of the phenotypic variability that typically characterizes bronchiolitis, with relevant implications for future research.

## Background

Bronchiolitis is the primary infection of the lower respiratory tract in children under 2 years of age, imposing a huge clinical burden as one of the commonest causes of hospital admission in this age group [1–4]. Although it is generally considered a single nosological entity, the clinical presentation could be extremely variable and recent studies have suggested that this condition is characterized by remarkable heterogeneity [5, 6]. Even hospitalized children may show various short-term outcomes (eg. length of hospital stay, risk of recurrence) [7–9], as well as different mid- and long-term sequelae, such as the risk of developing recurrent wheezing and asthma [10, 11]. Therefore, identifying different disease phenotypes might contribute to develop and implement proper preventive and therapeutic strategies. Most of the studies conducted so far have generally classified children with bronchiolitis on the basis of a limited number of characteristics (eg. type of virus, presence of wheezing, severity score). Of note, a multidimensional approach has proven to be more effective in clarifying the intrinsic heterogeneity of other respiratory disorders such as asthma and wheezing in childhood. In this context, clustering statistical methods are suitable for categorizing a heterogeneous population into subpopulations who share some aspects of a disease [12]. In particular, Latent Class Analysis (LCA) is a “hypothesis-free” approach, which assigns patients to classes based on their homogeneous characteristics, rather than them being arbitrarily assigned to classes by the researchers [13]. This method has been successfully used to detect subgroups of children with wheezing or asthma who share similar characteristics of the disease [14–17]. Using this statistical approach in multicenter cohorts of US (MARC-30 USA) and Finnish (MARC-30 Finland) children hospitalized with severe bronchiolitis, disease profiles were identified that differed based on the personal history of wheezing/eczema, wheezing during acute infection, severity levels and viral aetiology [18]. More recently, LCA has been applied in a study conducted on a large cohort of US children (MARC-35) hospitalized for bronchiolitis and followed up to the age of three, allowing the identification of three disease profiles significantly different with respect to inflammation and atopy markers, the nasopharyngeal microbiota, and the development of recurrent wheezing at 3 years of life [19].

No similar studies have been performed in the Italian children population so far. Therefore, the aim of the current study was to identify and describe discrete profiles of children hospitalized with bronchiolitis by means of LCA in a retrospective, single centre study conducted in Palermo, Italy, and to compare our findings with those obtained in international cohorts.

## Methods

### Study design and data collection

This is a retrospective observational study conducted on children aged ≤2 years hospitalized for bronchiolitis at the Department of Infectious Diseases and the University Department of General Pediatrics in “Giovanni Di Cristina” Pediatric Hospital of Palermo, Italy, between November 2012 and May 2019. Patients were identified based on a bronchiolitis diagnosis (ICD-10 code J21.0–9) at discharge. Patients’ medical files were reviewed to check that bronchiolitis diagnosis was in accordance with international guidelines [3] and data relevant to the study were analysed and reported anonymously, thus the ethical research committee approval was waived.

The following data were collected: gender, age, number of cohabitants in the home, number of siblings; mode of delivery, gestational age, birth weight, perinatal problems (need for resuscitation, O_2_ therapy, hospitalization, administration of surfactant, mechanical ventilation), prophylaxis with Palivizumab, breastfeeding history; underlying chronic diseases; clinical characteristics at admission: weight, respiratory rate, SpO_2_, body temperature, occurrence of respiratory, gastrointestinal and neurological symptoms and signs, feeding difficulties; chest radiological findings, type of virus identified in the nasal swab; treatment; number of days of hospitalization.

Viruses were detected in nasopharyngeal swabs by genome detection using reverse transcriptase polymerase chain reaction or by viral antigens detection using immunofluorescence or enzyme immunoassays. All samples were tested for the presence of RSV, RV, Enterovirus, Influenza virus A and B, Parainfluenza virus 1–4, human Coronavirus OC43, 229E, NL-63 and HUK1, Adenovirus, and Human Metapneumovirus.

### Statistical analysis

Data were presented as n (%) or mean (SD). Differences of categorical variables were analysed using Chi-squared test. LCA was used to discover underlying response patterns, thus allowing the identification of respondent groups with similar characteristics. LCA was computed using the R poLCA package, which estimates the latent class model by maximizing, with respect to *p*_*r*_ and *π*_*jrk*_, the following log-likelihood function:

$$ \ln L=\sum \limits_{i=1}^N\mathit{\ln}\sum \limits_{r=1}^R{p}_r\prod \limits_{j=1}^J\prod \limits_{k=1}^{K_j}{\pi_{jrk}}^{Y_{ijk}} $$

where J indicates polytomous categorical variables (manifest variables), each containing K_j_ possible outcomes, for individuals i = 1…N; *Y*_*ijk*_ denotes observed values of J manifest variables such that *Y*_*ijk*_ = 1 if the respondent *i* gives the k-th response to the j-th variable, and *Y*_*ijk*_ = 0 otherwise; *π*_*jrk*_ denotes class-conditional probability that an observation in class r = 1….R produces the k-th outcome on the j-th variable and *p*_*r*_ indicates R mixing proportions. poLCA takes advantage of the iterative nature of the expectation-maximization algorithm to make it possible to estimate the latent class model even when some of the observations on the manifest variables are missing.

The method assumes that all associations between the included variables are entirely due to the existence of distinct subpopulations called latent classes. Within the latent classes all variables are assumed to be independent [20]. The responses to these items (i.e. manifest variables) were used to categorize respondents into groups with similar response profiles (i.e. latent classes). Variable selection for LCA was performed in order to find the set of variables with relevant clustering information and to discard those that were redundant and/or not informative. For performing variable selection, we used LCAvarsel [21] function with forward search strategy. The algorithm starts from the minimum subset of variables that allows a latent class analysis model to be identified, then the variables are added/removed in turn to/from the set of clustering variables until no further change to the set of selected ones. The initial set of clustering variables is chosen by default using the strategy described in Dean and Raftery [22]. The final model included gender, age (< 6 months or ≥ 6 months), RSV infection (yes or no), respiratory distress (yes or no), apnea (yes or no), and cough (yes or no). The Akaike Information Criterion (AIC) [23] was computed in order to select the best number of classes by comparing models with one to five classes.

AIC derived from LCAs suggested that a three-class model was favoured. Robustness of our results was tested by fitting LCA excluding children ≥12 months old with findings substantially unchanged (Fig. S1). Analyses were performed using R 3.5.2 software. A *p*-value < 0.05 was considered statistically significant.

## Results

### Characteristics of study population

A total of 401 children were enrolled. Table 1 reports demographic, perinatal and neonatal characteristics of the study population. Fifty-five children were preterm born. Three children were diagnosed with a chromosomic syndrome and ten had congenital heart diseases. One child had intestinal atresia, one had congenital hypothyroidism and another one had a diagnosis of epilepsy. Out of the 55 preterms enrolled in the study, two were affected by Down syndrome and three had congenital heart disease.

**Table 1:** Demographic, perinatal and neonatal characteristics of the study population

	*n* = 401
Gestational age, weeks	38.25 (2.86)
Preterm birth (gestational age < 37 weeks)	55 (13.7)
Birth weight, Kg	3.07 (0.60)
Singleton delivery	390 (97.25)
Type of delivery	
Elective caesarean section	113 (30.38)
Emergency caesarean section	43 (11.56)
Vaginal	216 (58.06)
O_2_ therapy at birth	33 (8.23)
Resuscitation	23 (5.74)
Surfactant at birth	4 (1.00)
Neonatal hospitalization	47 (11.72)
Mechanical ventilation	11 (2.74)
First-born	162 (40.40)
Number of siblings	0.91 (1.09)
Number of cohabitants	3.74 (1.29)
Birth within 3 months of the epidemic season start	22 (5.49)
Breastfeeding (exclusively)	108 (37.24)
Breastfeeding duration, months	0.61 (1.71)
Palivizumab prophylaxis	2 (0.50)

Data are presented as n (%) or mean (SD)

Table 2 reports demographic, clinical, microbiological and radiological findings at hospitalization. 59.1% of the study sample was represented by male patients; 70.32% were aged less than 6 months at the time of admission. The most frequently observed clinical findings were: cough (83.46%), respiratory distress (53.94%) and rhinorrea (34.61%). All children underwent nasal swabs for microbiological diagnosis. In 53.61% viruses could not be identified; in 44.13% a single virus was detected and in 2.24% a double viral infection was found. The most common viruses detected in nasal swabs were: RSV (35.91%), Parainfluenza virus (6.23%) and Influenza virus (2%). Chest X-ray was performed in 56.86% of patients and pneumonia was the most frequently observed finding (30.67%).

**Table 2:** Demographic, clinical, microbiological and radiological findings at hospitalization

Gender, male	237 (59.1)
Age, months	4.70 (4.93)
Weight, Kg	6.41 (2.49)
Clinical findings	
RR (breaths/min)	50.28 (11.19)
SpO_2_, %	96.04 (3.35)
Fever	3 (0.76)
Rhinorrea	136 (34.61)
Cough	328 (83.46)
Wheezing	3 (0.76)
Apnoea	16 (4.07)
Respiratory distress	212 (53.94)
Cyanosis	15 (3.82)
Feeding difficulties	20 (5.09)
Hypotonia	1 (0.25)
Seizures	1 (0.25)
Microbiological findings	
None	215 (53.61)
One virus	177 (44.13)
Co-infections (≥2 viruses)	9 (2.24)
VRS	144 (35.91)
Parainfluenza	25 (6.23)
Influenza	8 (2.00)
Rhinovirus	2 (0.50)
Adenovirus	3 (0.75)
Coronavirus	2 (0.50)
Enterovirus	1 (0.25)
Chest X-ray findings	228 (56.86)
Bronchitis	195 (23.69)
Pneumonia	123 (30.67)
Atelectasis	1 (0.25)

Data are presented as n (%) or mean (SD)

### Latent class analysis

**Fig. 1:**
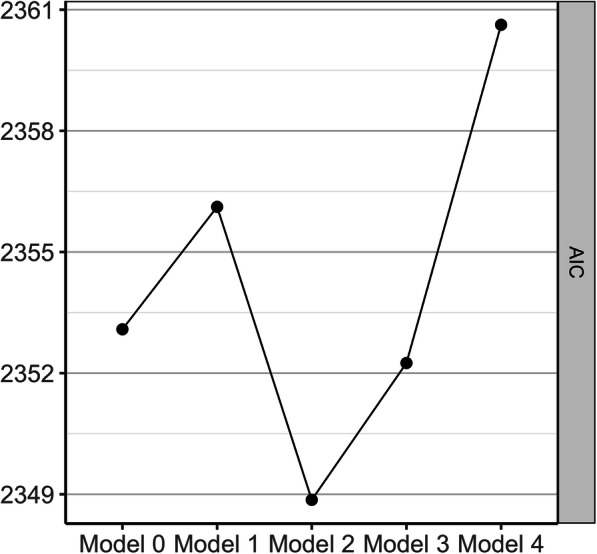
AIC of different latent class models. The AIC derived from LCA suggested that a three-class model was favoured by the lowest AIC

Five models were estimated from Model 0 (with only one class) to Model 4 (with five classes). The AIC derived from LCA suggested that a three-class model was favoured by the lowest AIC (Fig. 1).

**Fig. 2:**
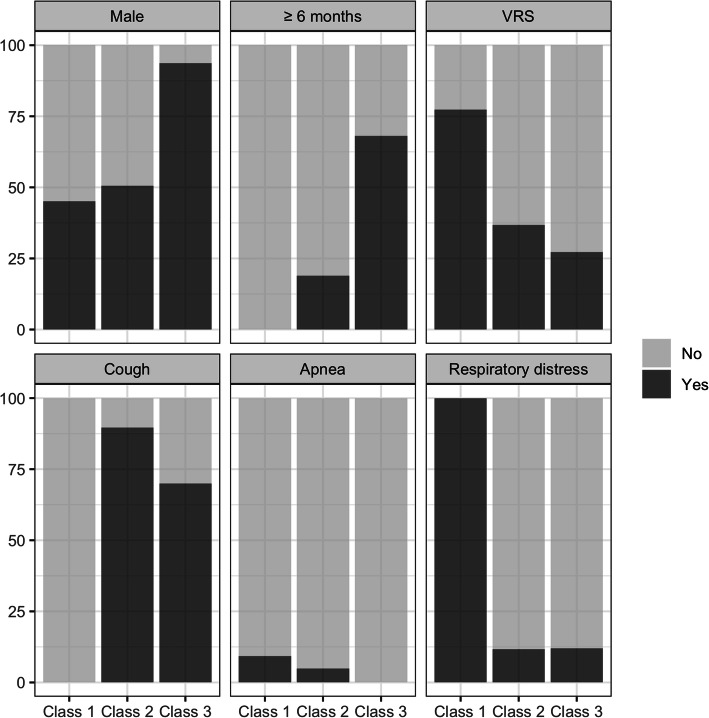
Response probabilities among the three latent classes. The figure illustrates the three classes identified by means of LCA

Figure 2 illustrates the three classes identified by means of LCA.

Class 1 (*n* = 14, 3.49%) was composed of 45% male children; all children in this class were aged ≤6 months at hospitalization; most of them were infected with RSV (77%); 100% had respiratory distress, 11% had apnea and none had cough.

Class 2 (*n* = 309, 77%) was predominantly composed of male children (51%); 19% were aged > 6 months at admission; 37% were infected with RSV; 12% had respiratory distress, 5% had apnea and 90% had cough.

Class 3 (*n* = 78, 19%) was characterized by the highest proportion of male children (94%); 68% were aged > 6 months at hospitalization; 27% were infected with RSV; 12% had respiratory distress, none had apnea and 70% had cough.

Table 3 compares demographic, perinatal and neonatal characteristics among the three latent classes. Children in Class 1 were more frequently born near the epidemic season (*p* = 0.028); breastfeeding duration was significantly longer for children in Class 3 (*p* = 0.004). Children in Class 2 were more frequently treated with systemic corticosteroids (*p* < 0.001), and low-flow oxygen therapy (*p* = 0.042). Children in Class 3 more frequently were administered with systemic antibiotics (*p* = 0.013).

**Table 3:** Demographic, perinatal and neonatal characteristics of the study population by LCs

	Class 1	Class 2	Class 3	*P*-value
	*n* = 14	*n* = 309	*n* = 78	
Age at hospitalization (≥6 months)	0 (0.00)	34 (11.26)	78 (100.00)	< 0.001
Gestational age, weeks	38.79 (1.97)	38.23 (3.02)	38.23 (2.27)	0.775
Birth weight, Kg	3.05 (0.48)	3.10 (0.60)	2.97 (0.60)	0.339
Singleton delivery	14 (100.00)	301 (97.41)	75 (96.15)	0.678
Type of delivery				0.534
Elective caesarean section	4 (28.57)	91 (31.71)	18 (25.35)	
Emergency caesarean section	2 (14.29)	29 (10.10)	12 (16.90)	
Vaginal	8 (57.14)	167 (58.19)	41 (57.75)	
O_2_ therapy at birth	2 (14.29)	22 (7.12)	9 (11.54)	0.314
Resuscitation	1 (7.14)	15 (4.85)	7 (8.97)	0.366
Surfactant at birth	0 (0.00)	3 (0.97)	1 (1.28)	0.902
Neonatal hospitalization	2 (14.29)	38 (12.30)	7 (8.97)	0.685
Mechanical ventilation	1 (7.14)	7 (2.27)	3 (3.85)	0.441
First-born	4 (28.57)	121 (39.16)	37 (47.44)	0.271
Number of siblings	0.93 (0.83)	0.96 (1.13)	0.73 (0.91)	0.256
Number of cohabitants	3.64 (1.45)	3.79 (1.26)	3.55 (1.35)	0.322
Birth within 3 months of the epidemic season start	3 (21.43)	15 (4.85)	4 (5.13)	**0.028**
Breastfeeding (exclusively)	1 (9.09)	79 (35.75)	28 (48.28)	0.113
Breastfeeding duration, months	0.21 (0.80)	0.48 (1.63)	1.18 (2.04)	**0.004**
Palivizumab prophylaxis	0 (0.00)	2 (0.64)	0 (0.00)	–
Hypertonic saline	1 (7.14)	5 (1.62)	0 (0.00)	0.120
Inhaled bronchodilators	6 (42.86)	157 (50.81)	46 (58.97)	0.339
Inhaled corticosteroids	9 (64.29)	233 (75.40)	66 (84.62)	0.120
Systemic corticosteroids	12 (85.71)	282 (91.26)	55 (70.51)	**< 0.001**
Systemic antibiotics	7 (50.00)	203 (65.70)	63 (80.77)	**0.013**
Low-flow oxygen therapy	6 (42.86)	144 (46.60)	24 (30.77)	**0.042**
Length of hospital stay	9.21 (7.93)	7.75 (18.42)	5.17 (3.10)	0.415
Intensive care admission	2 (14.29)	38 (12.30)	7 (8.97)	0.685

Data are presented as n (%) or mean (SD)

## Discussion

The current study identified distinct clinical profiles of children hospitalised for bronchiolitis through the application of a hypothesis-free statistical clustering approach, such as LCA, which allowed us to describe three discrete phenotypes of patients based on gender, age, clinical characteristics at admission.

Clustering statistical methods are useful for identifying profiles that summarize shared aspects of disease within different groups of patients. LCA has been previously used to identify three to four homogeneous subgroups of patients starting from a large series of observed characteristics in multicenter cohorts of hospitalized children with severe bronchiolitis [18]. More recently, the same approach has been applied in a study conducted on a large population of US children hospitalized for bronchiolitis and followed up to the age of 3 years, finding three distinct profiles of children that showed significant differences with respect to markers of inflammation and atopy, nasopharyngeal microbiota, and respiratory outcomes by 3 years of age [19]. To our knowledge, no previous studies have identified classes of children with bronchiolitis within the Italian population. Noteworthy, the bronchiolitis profiles identified in the current study by means of LCA share some similarities with those identified in the aforementioned studies using the same statistical approach.

We identified a group of children (Class 1) predominately composed of female subjects and characterized by early onset (< 6 months of age) of symptoms. In this group, bronchiolitis was mainly caused by RSV (77%); all patients had respiratory distress, and apnoea episodes were present in 11%. These children share similarities with Profile C children identified in the two US cohorts (MARC-30 USA and MARC-35), in which patients showed the lowest age at admission, had the most severe clinical presentation and were mostly infected by RSV (> 80%). Conversely, in the Finnish cohort (MARC-30 Finland), children with a more serious clinical presentation (Profile A) were mostly aged ≥12 months and had an RV infection in 54%. Interestingly, Profile C children in the MARC-35 cohort showed an augmented risk of recurrent wheezing by age 3 years. This result confirms that the severity of bronchiolitis may influence mid-term respiratory outcomes, increasing the risk of recurrent wheezing and asthma later in life [24–26].

The largest group of children identified in the current study (Class 2) was mainly composed of male subjects; 19% were aged > 6 months at the time of hospitalization and 37% were infected by RSV. The majority of children (90%) had cough at presentation; 12% showed respiratory distress and 5% presented with apnoea. This class, characterized by an intermediate level of severity, shared similarities with Profile B children in the MARC-35 cohort and with Profile BC in the MARC-30 Finland cohort, both characterized by a prevalence of male subjects, which however showed a younger age at the time of hospitalization and were predominantly infected by RSV. On the other hand, no similarities could be detected with the bronchiolitis profiles identified in the MARC-30 USA cohort, probably due to the greater number of patients (*n* = 2207) which justifies an even more marked phenotypic heterogeneity in this population.

Class 3 included the largest proportion of male subjects (94%) and was mostly composed of children aged > 6 months at the time of admission (68%). 70% of children had cough, 12% showed respiratory distress and none presented with apnoea. Similarly to Profiles D identified in MARC-30 USA and MARC-30 Finland cohorts, this was the least severely ill group, and it differed from other classes by the lowest proportion of children with RSV infection (27%), which was in agreement with Profile A in the MARC-35 cohort. Of note, also these profiles were mainly composed of male subjects and, with the exception of the MARC-30 USA cohort, they were characterized by a higher proportion of children aged > 6 months.

With regard to the statistically different characteristics for the three classes identified in the current study, we found that children in Class 1 were more frequently born near the epidemic season. This is considered a risk factor both for the development of lower respiratory tract infections as well as for hospitalization in subjects with RSV infection, who likely develop more frequently severe symptoms if born during the first half of the epidemic season [27]. Together with the low age at the time of hospitalization (< 6 months) and the higher prevalence of infections by RSV, this may have contributed to the greater severity of clinical presentation observed in children in Class 1 compared to the other two classes. Indeed, evidence suggests that the RSV aetiology is associated with greater severity of the clinical presentation [28–30] and that a lower age at admission is associated with greater clinical severity of the RSV infection [28, 31].

As a piece of further evidence derived by the comparison of the three classes identified in the current study, duration of exclusive breastfeeding was significantly longer in children in Class 3, which was characterized by the least clinical severity of the disease. Notably, the differences among LCA classes in breastfeeding duration are due to the highest percentage in Class 3 of children who were exclusively breastfed. Indeed, when children who were not exclusively breastfed are excluded similar duration of breastfeeding are found. Thus, duration of breastfeeding can be considered as a proxy of exclusive breastfeeding. Even though the length of exclusive breastfeeding observed in this study was on average lower than 6 months or even 4 months, our finding provides further evidence for a protective role of breastfeeding against the severity of bronchiolitis, probably due to immunomodulatory factors in human milk against the RSV, as suggested by previous studies [32, 33].

The application of a statistical methodology such as LCA allowed us to detect bronchiolitis profiles different in gender, age at the onset of symptoms, severity of the clinical picture. These profiles were obtained directly from the data collected within a heterogeneous sample of children rather than being arbitrarily assigned. The resulting classes show a certain degree of flexibility: each individual can be assigned to different classes with different probabilities and this classification reflects clinical reality more closely, emphasizing the effectiveness of a multidimensional approach in capturing the clinical heterogeneity of the disease. A possible drawback is given by the need to establish a priori the set of variables, which requires a certain degree of subjectivity in data analysis. A further limitation of our study is related to the identification of disease profiles in a cohort of hospitalized children, which are actually a small proportion of all children with bronchiolitis. For a better characterization of the disease, it could be useful to apply the same statistical approach also to children with a milder level of disease, not requiring hospitalization, as they could show different socio-demographic characteristics and risk factors, as well as different clinical characteristics, compared to patients with most severe cases. However, our study allowed us to highlight that even within the group of children with bronchiolitis who require hospitalization there is a certain level of heterogeneity that could influence the diagnostic and therapeutic approach. Furthermore, the choice to study this cohort of children is significant, given that bronchiolitis is the most frequent cause of hospitalization in infants [34] and that patients with more severe disease have a higher risk of developing respiratory sequelae later in life. Lastly, it should be acknowledged as a limitation that viruses could not be identified in more than 50% of the enrolled patients, thus our results should be interpreted with caution with respect to viral aetiology. Indeed, our virus detection rate was poor, likely due to the different diagnostic tests used (immunofluorescence/enzyme immunoassays vs. PCR) as well as to technical problems in collecting and storing biological samples. Nonetheless, our detection rate is quite similar to those reported in other epidemiologic studies in Italy [35, 36].

## Conclusions

Describing profiles of a heterogeneous disease such as bronchiolitis could be useful in providing the appropriate clinical approach and in identifying the best available therapeutic choice. The current findings may help to increase the understanding of the phenotypic variability that typically characterizes bronchiolitis, although they need validation in further studies. Namely, prospective longitudinal studies are needed to define bronchiolitis phenotypes and endotypes through an integrated clinical, epidemiological and molecular approach in order to develop personalized clinical management and clarify the physiopathologic basis of mid- and long-term respiratory outcomes [6].

## Supplementary information

**Additional file 1: Figure S1.** Response probabilities among the three latent classes excluding children aged ≥12 months.

## Data Availability

The datasets used and/or analysed during the current study are available from the corresponding author on reasonable request.

## Abbreviations

AIC: Akaike Information Criterion
LCA: Latent Class Analysis
RSV: Respiratory syncytial virus
RV: Rhinovirus
