# Supplementary material for: Identification of bronchiolitis profiles in Italian children through the application of latent class analysis
Source: Ital J Pediatr. 2020 Oct 7;46:147. doi: 10.1186/s13052-020-00914-4 (PMC7539284; doi:10.1186/s13052-020-00914-4)

**Figure S1.** Response probabilities among the three latent classes excluding children aged ≥12 months.


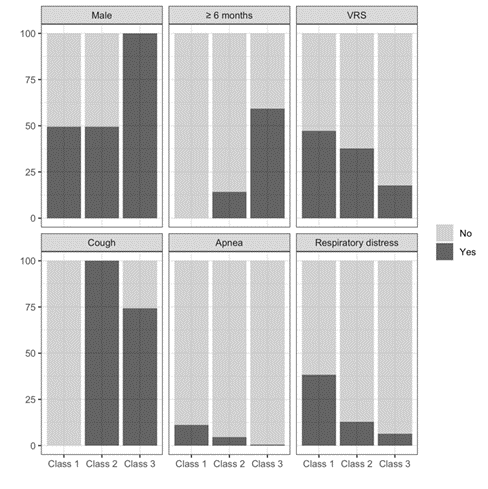

Supplement: Supplementary file 1 — Additional file 1: Figure S1. Response probabilities among the three latent classes excluding children aged ≥12 months. [file 13052_2020_914_MOESM1_ESM.docx]
